# Copper Homeostasis, Emerging Central Players in Cancer Immunotherapy

**DOI:** 10.1111/jcmm.70611

**Published:** 2025-10-13

**Authors:** Chengxin Chen, Mengle Peng, Shuhong Liang, Chunwei Li, Lili Zhu, Yaqi Yang, Lifeng Li, Wenhua Xue

**Affiliations:** ^1^ Department of Pharmacy The First Affiliated Hospital of Zhengzhou University Zhengzhou China; ^2^ Department of Clinical Laboratory Henan No. 3 Provincial People's Hospital Zhengzhou China; ^3^ National Engineering Laboratory for Internet Medical Systems and Applications, the First Affiliated Hospital of Zhengzhou University, Zhengzhou University Zhengzhou China; ^4^ Medical School Huanghe Science and Technology University Zhengzhou China; ^5^ Cancer Center The First Affiliated Hospital of Zhengzhou University Zhengzhou China

**Keywords:** cancer, copper homeostasis, cuproptosis, immunotherapy, mitochondria

## Abstract

Copper ions serve as multifunctional signalling molecules that are integral to various cellular processes in vivo, including cell proliferation, apoptosis and migration. The storage, transport and homeostatic regulation of intracellular copper are closely linked to mitochondrial function. These biological processes play a significant role in the occurrence and development of tumours. Furthermore, cuproptosis, a newly identified mode of cell death, has been associated with multiple cellular processes, such as mitochondrial respiration, antioxidant defence, redox signalling, kinase signalling, autophagy and protein quality control. In this study, we investigate the relationship between dysregulation of mitochondrial‐centered copper homeostasis in cancer and the activation of the NF‐κB signalling pathway, as well as the transcriptional regulation of PD‐L1. Our findings reveal a potential connection between copper ion signalling and the activation of anti‐tumour immunity. We outline the advantages and challenges of copper‐targeted anticancer therapeutic strategies and emphasise the necessity for a deeper understanding of copper homeostasis regulation in cancer. We review the evidence of altered copper homeostasis in tumours, discuss the current understanding of the mechanisms underlying these changes, and consider the potential implications for cancer progression. The imbalance of copper in tumours may present opportunities for the development of novel imaging biomarkers and therapeutic agents.

**Abbreviations**
AD: Alzheimer's disease
AKT: Protein Kinase B
ALS: Amyotrophic lateral sclerosis
ATOX1: Antioxidant 1 Copper Chaperone
ATP7A: ATPase Copper Transporting Alpha
ATP7B: ATPase Copper Transporting Beta
BAX: BCL2 Associated X, Apoptosis Regulator
CASP1: Caspase 1
CP: Ceruloplasmin
DDC: Dithiocarbamate
DLAT: Dihydrolipoamide S‐Acetyltransferase
DSF: Disulfiram
EMT: Epithelial‐Mesenchymal Transition
ER: Endoplasmic reticulum
ERK1/2: Mitogen‐Activated Protein Kinase 3/1
FABP7: Fatty Acid Binding Protein 7
FDX1: Ferredoxin 1
GSDMD: Gasdermin D
GSH: Glutathione
HIF1A: Hypoxia Inducible Factor 1 Subunit Alpha
ICD: Immunogenic cell death
IL‐17: Interleukin 17A
JNK: C‐Jun N‐terminal kinase
LIAS: Lipoic Acid Synthetase
LOX: Lysyl Oxidase
MEK1/5: Mitogen‐Activated Protein Kinase 1/5
MMP2: Matrix Metallopeptidase 2
PI3K: Phosphatidylin‐ositol‐3‐kinase
PMAIP1/NOXA: Phorbol‐12‐Myristate‐13‐Acetate‐Induced Protein 1
ROS: Reactive oxygen species
SCO1/2: Synthesis Of Cytochrome C Oxidase 1/2
SLC31A1 (CTR1/HCTR1): Solute Carrier Family 31 Member 1
SLC46A3: Solute Carrier Family 46 Member 3
SLC7A11: Solute Carrier Family 7 Member 11
STAT3: Signal Transducer And Activator Of Transcription 3
STEAP: STEAP Family Member 1
TGN: Trans‐Golgi apparatus network
TM: Tetrathiomolybdate
VEGF: Vascular Endothelial Growth Factor A

## 1 Introduction

As an essential trace element, copper ions play a crucial role in various biological processes within the body, including cell proliferation, apoptosis, migration and immune response [1, 2]. While the significance of Cu^2+^ regulation in cancer has long been recognised, recent research has brought renewed attention to this area [3, 4]. The discovery of cuproptosis, a novel form of cell death, has provided compelling evidence that dysregulated copper homeostasis is a key factor in cancer initiation and progression, as well as impacting treatment outcomes for cancer patients [5, 6, 7]. Consequently, Cu^2+^ signalling has emerged as a promising target for the development of new anticancer drugs [8, 9].

Copper ions (Cu^2+^) play a crucial role in regulating cellular life activities due to the stringent control exerted by a complex network of components. This network encompasses various molecular interactions, including Cu^2+^ carriers, channel proteins and signalling molecules [10, 11]. Advances in metal imaging techniques in biology have elucidated the spatial distribution of copper within cells and organisms [12]. The cellular mechanisms maintain the cytoplasmic concentration of copper ions at nearly zero levels [13]. Predominantly, copper exists in the cytoplasm in the form of complexes. These complexes can be categorised into two distinct pools: the static pool, where the metal is tightly bound to proteins and other macromolecules, and the unstable pool, where the metal is weakly bound to cellular ligands, exhibiting mobile characteristics [14, 15]. When Cu^2+^ enters the cytoplasm in a free state, it is reduced by acquiring an electron, resulting in the formation of positively charged organic free radicals, which may subsequently contribute to the generation of reactive oxygen species (ROS) [16]. ROS are known to be detrimental to cellular components, including proteins, lipids and nucleic acids, and have been associated with aging, neurological diseases and cancer [17, 18].

Interestingly, comparative studies of fibroblasts lacking the mitochondrial copper metallochaperones SCO1 and SCO2, as well as the copper export pump ATP7A, have shown that total and labile mitochondrial copper pools remain constant even when whole‐cell copper pools are altered [19, 20]. Under oxidative stress, reactive oxygen species (ROS) produced by mitochondria serve as major mediators of tumorigenesis, influencing various aspects such as proliferation, migration/invasion, angiogenesis, inflammation and immune evasion, thereby allowing cancer cells to adapt to a challenging environment [21]. These findings reveal that copper homeostasis plays a crucial role in mitochondrial function and significantly impacts cancer progression. In this context, we focus on our recent understanding of the regulation of mitochondrial‐centered Cu^2+^ homeostasis and its relationship with cancer immunotherapy.

## 2 Achieving Organelle Copper Homeostasis

### 2.1 Intracellular Copper Ion Transport and Mitochondrial Function

Mitochondria, as crucial organelles in eukaryotic cells, play a significant role in energy production, biotransformation of amino acids and lipids, as well as the regulation of apoptosis and autophagy [22, 23]. There exists an inseparable relationship between copper homeostasis and mitochondrial function, making it essential to understand the mechanisms by which copper ions target mitochondria within cells [24]. Numerous studies have demonstrated that the homologous trimer CTR1 serves as a vital channel protein that regulates the transport of copper ions into cells [25]. However, this channel may impose stringent requirements on the valence state of copper ions; research indicates that the channel protein exhibits high specificity for Cu^1+^. Consequently, the potential mechanism for Cu^2+^ transport into cells could involve its reduction to Cu^1+^ by steap protein, enabling its entry through CTR1 [26, 27].

**FIGURE 1:**
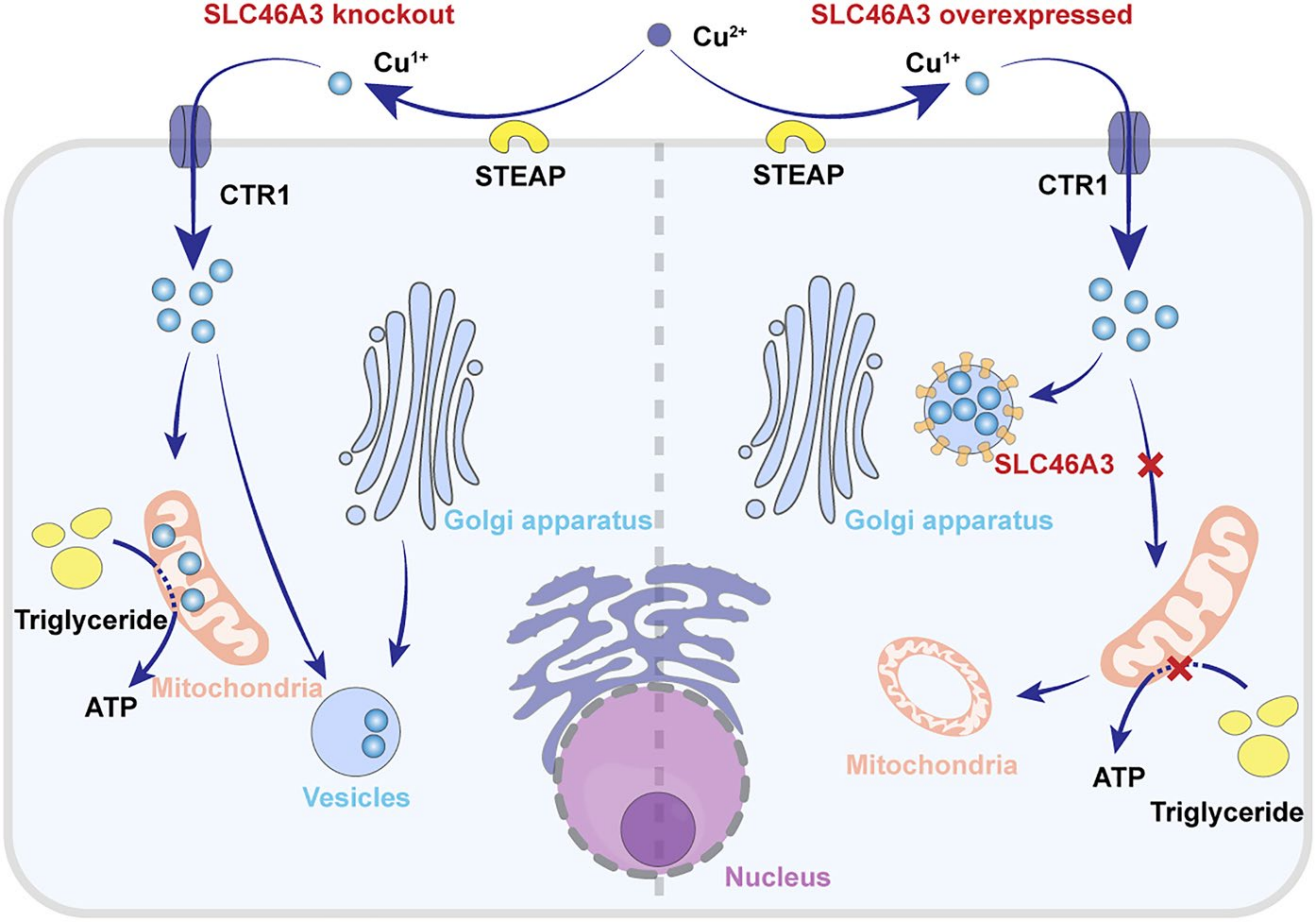
The mechanism of SLC46A3 regulating copper homeostasis in cells.

The translocation of copper to mitochondria may engage one or more intracellular ligands that have yet to be characterised in detail. These ligands facilitate the transport of copper ions to the mitochondrial membrane gap [27]. Copper ions directly influence mitochondrial respiratory complex IV and cytochrome c oxidase; thus, a copper deficiency in cells may result in mitochondrial dysfunction [23]. Recent findings suggest that SLC46A3, a member of the solute carrier family, can induce mitochondrial dysfunction by causing intracellular copper deficiency [28]. Overexpression of SLC46A3 leads to intracellular copper deficiency, which inhibits mitochondrial potential and can even alter mitochondrial morphology [28]. Therefore, the impact of intracellular copper homeostasis on mitochondria is profound, offering new insights into the mechanisms underlying metabolic reprogramming in cancer. The specific mechanisms through which SLC46A3 influences copper metabolism and subsequently alters mitochondrial function are illustrated in Figure 1.

In recent years, aerobic glycolysis has been widely recognised as the primary energy source for tumours, a phenomenon known as the Warburg effect. However, the discovery that mitochondrial metabolism serves as a significant energy source for certain cancer cells has led to increasing evidence suggesting that both mitochondrial energy supply and aerobic glycolysis play equally crucial roles in the development of cancer cells.

### 2.2 Regulation of Lysosomal and Golgi Transport on Intracellular Copper Homeostasis

Lysosomes play a crucial role in the transport of copper from cells to mitochondria. The significance of lysosomes and the Golgi apparatus in maintaining intracellular copper homeostasis is gaining increasing recognition [29]. Lysosomes are catabolic organelles in eukaryotic cells that digest various cytoplasmic components. Previous reports have indicated that copper can be transported to liver cells via a vesicular pathway and subsequently excreted into bile through lysosomes [30]. Recent studies have demonstrated that the expression of SLC46A3 increases the abundance of lysosomes, although the underlying mechanism remains unclear. Copper ions can be chelated in lysosomes through SLC46A3, and the deletion of SLC46A3 may result in alterations in mitochondrial morphology and membrane potential [28]. Copper ions directly bind to lipoylated enzymes (such as DLAT, DLST) in the mitochondrial TCA cycle, leading to abnormal enzyme aggregation and mitochondrial dysfunction. FDX1, as a key regulator of cuproptosis, determines the sensitivity of cells to cuproptosis through its expression level. The copper chaperone COX17 and the transporter ATP7A influence cuproptosis by regulating the subcellular localization of copper ions (such as mitochondria, Golgi apparatus) [31]. Elesclomol, a copper ionophore, induces cuproptosis and releases damage‐associated molecular patterns (DAMPs), which can activate dendritic cells and CD8^+^ T cells [32]. Additionally, studies have shown that the level of lipoylated DLAT fragments in the blood is positively correlated with cuproptosis activity. This marker can be used for real‐time monitoring of cancer patients' response to cuproptosis‐targeted therapy.

**FIGURE 2:**
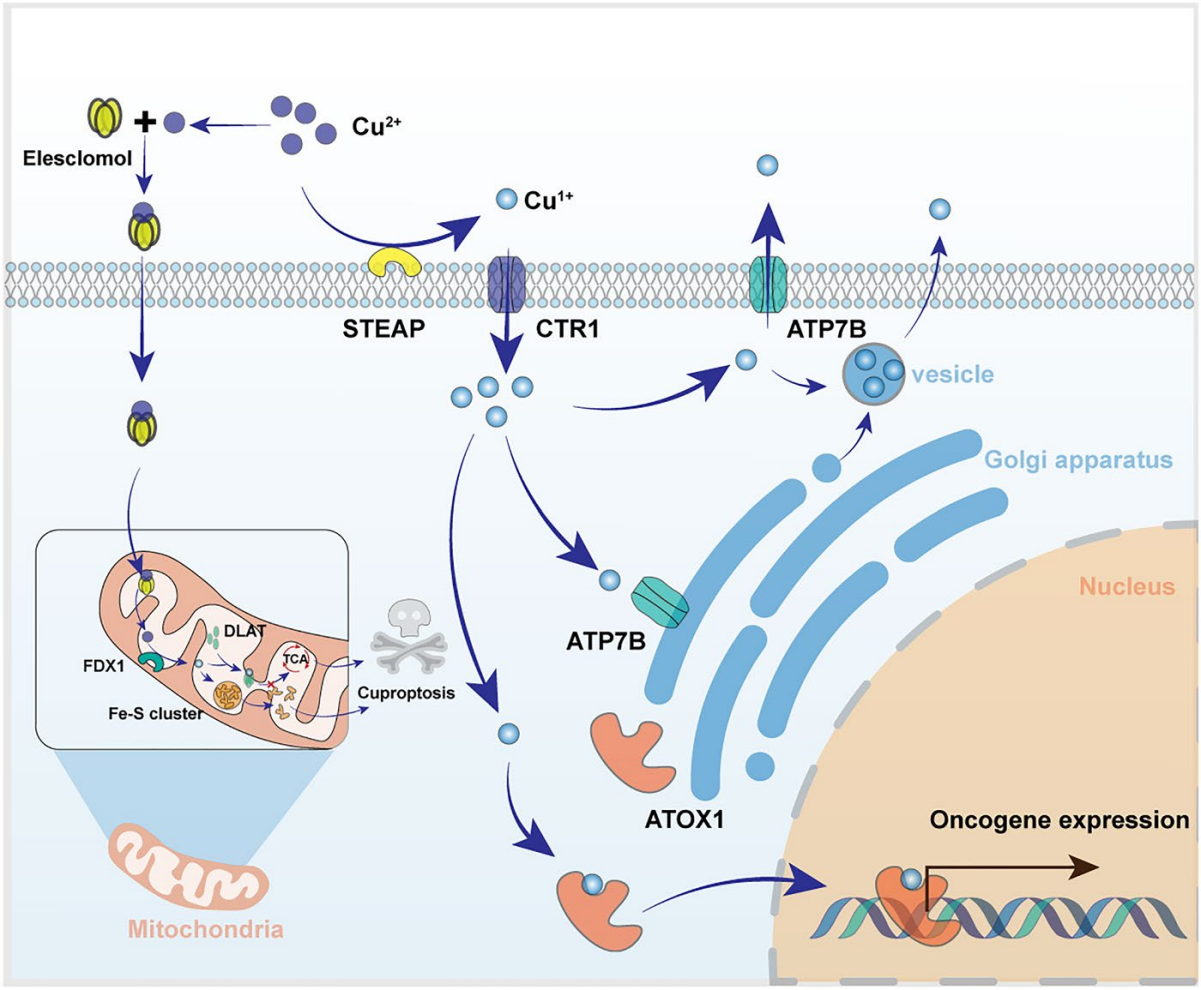
Graphical overview of copper steady‐state research results. Copper ions enter cells primarily through two mechanisms: the first involves direct binding with ethylchlorophenol, facilitating their entry into the cell; the second involves reduction by the STEAP protein located on the cell membrane, allowing copper ions to enter through the CTR1 copper ion channel. Copper ions bound to ethylphenol are subsequently transported to the mitochondria, where they play a role in regulating pyruvate kinase function. When free copper ions enter the cell, they participate in the regulation of electron transfer within the respiratory chain and influence nuclear gene transcription by binding with ATOX1. Most copper ions are transported to the Golgi apparatus via the ATP7B channel within the cell before being sent to extracellular vesicles.

Copper ions are vital for maintaining mitochondrial membrane potential, indirectly supporting the notion that lysosomes are essential for intracellular copper transport [33]. Additionally, ATP7A and ATP7B in the Golgi apparatus also participate in the copper transport process [34, 35]. Under normal conditions, extracellular copper ions enter cells via the copper transporter CTR1, bind to ATOX1, and are transported to intracellular vesicles produced by the Golgi apparatus through ATP7A or ATP7B, thereby maintaining copper homeostasis both intracellularly and extracellularly [36]. Excess copper is transported through vesicles and expelled from cells via exocytosis. The accumulation of copper ions in cells is often a significant factor in the occurrence and progression of various cancers. Increasingly, studies are focusing on the critical roles of lysosomes and the Golgi apparatus in these processes, providing new insights and strategies for cancer treatment [37]. The detailed regulatory mechanisms of copper ion homeostasis within cells are illustrated in Figure 2.

## 3 Disturbed Organelle Cu^2+^ Homeostasis and Tumorigenesis

### 3.1 Disturbed Organelle Copper Homeostasis and Cell Proliferation

The relationship between copper and tumour development has been recognised for many years. Numerous observations indicate that tumours require higher levels of copper compared to normal tissues [38]. During mammalian growth and development, copper is absorbed through the gastrointestinal tract and excreted via bile to maintain systemic copper homeostasis [39]. Once the growth stage is completed, copper homeostasis in the body reaches equilibrium. However, when the demand for anabolism increases, a higher level of copper is necessary [40]. This increased requirement is partly due to copper's role as a cofactor for mitochondrial cytochrome c oxidase, which is essential for the energy metabolism required for rapid cell division [41]. Consequently, cancer cells must achieve unlimited proliferation, leading to a greater demand for copper compared to non‐dividing cells.

In addition, copper is involved in the synthesis of a wide range of key biological processes, including respiration, neuropeptide maturation, protection from oxidative stress, angiogenesis and numerous other essential biological functions [42]. These processes are critical for tumour cell growth. Antioxidant‐1 (ATOX1) is a copper‐binding protein that facilitates the transport of copper to the secretory compartment via cytosolic copper chaperones, including the trans‐Golgi apparatus network (TGN) [36]. More than a decade ago, ATOX1 was identified as being expressed in the nuclei of arteriosclerotic neointimal lesions, which contain highly proliferative cells. Similarly, 45% of the pups of ATOX1‐deficient mice died before weaning, and the surviving animals exhibited severe growth disorders [43]. Copper also plays a significant role not only in energy metabolism but also in the induction of inflammation. The metal reductase STEAP4 is crucial for the valence change and transportation of copper within cells. It has been observed that inflammatory cytokines, such as IL‐17, promote cellular copper uptake by inducing the expression of metal reductase STEAP4 [44]. Consequently, the IL‐17–STEAP4–XIAP signalling axis is activated, leading to increased copper uptake that promotes the development of colon tumours [45]. These findings underscore the indispensable role of copper ions in both normal cell growth and tumour cell proliferation.

Given the significant role of copper ions in the proliferation of cancer cells, a series of drugs targeting copper ion transport have been developed in recent years, demonstrating substantial therapeutic effects in inhibiting cancer proliferation. One of the most prominent approaches is the application of copper ion chelating agents. For instance, tetrathiomolybdate (TM) [46, 47], a specific copper chelator, has been shown to effectively reduce copper levels in cells, thereby significantly inhibiting tumour angiogenesis and growth. Additionally, methods that induce oxidative stress‐induced tumour cell death through copper complexes that transport copper into mitochondria have also been reported. Elesclomol, as a representative example, has demonstrated efficacy in treating various cancers [48]. However, most studies indicate that the drug's tumour‐suppressive mechanism primarily involves copper‐induced oxidative stress within mitochondria, leading to cancer cell death. The introduction of the concept of cuproptosis has provided a more precise explanation of how this drug inhibits tumour occurrence and progression [5]. Unlike previous perspectives that focused on changes in mitochondrial membrane potential and oxidative stress, cuproptosis connects copper ions with the tricarboxylic acid cycle and the production of reactive oxygen species in mitochondria [49]. This insight offers a novel perspective and target for cancer treatment strategies aimed at inhibiting cancer cell proliferation.

### 3.2 Disturbed Organelle Copper Homeostasis and Cancer Metastasis

Tumour cell migration remains a significant challenge that impedes the efficacy of cancer treatments and is closely linked to increased mortality among cancer patients [14]. Notably, it has been observed for some time that treatment with TM (a copper ion chelating agent) markedly reduces the mobility and invasiveness of head and neck tumour cells by inhibiting the activity of lysine oxidase (LOX) [50], Focal Adhesion Kinase (FAK) activation, and the level of Matrix Metalloproteinase 2 (MMP2) [51]. However, the relationship between copper transporters and LOX family members has not yet been adequately explored. Recent studies indicate that Atox1 mediates the migration of breast cancer cells through a coordinated copper transport mechanism involving the ATP7A–LOX axis [52]. Given that single‐cell migration is an early step in breast cancer metastasis, the level of Atox1 in tumour cells may serve as a predictive indicator of metastatic potential. This study elucidates the critical relationship between copper and breast cancer metastasis. Furthermore, related clinical studies have compellingly demonstrated that copper depletion significantly reduces lung metastasis in triple‐negative breast cancer. Similarly, lysine oxidase‐like 2 (LOXL2) is a copper‐dependent enzyme belonging to the lysine oxidase family, which promotes the reorganisation of the cytoskeleton and the invasion of oesophageal cancer cells by interacting with cytoplasmic actin‐binding proteins, such as ezrin [53]. Previous studies have shown that LOXL2 enhances cell migration and invasion, stimulates the formation of filopodia, regulates the expression of cytoskeletal genes, and promotes tumour development and metastasis in vivo. Recently, a study demonstrated that ATP7A is essential for the activity of multiple members of the copper‐dependent lysyl oxidase LOX family, and silencing ATP7A in two different cancer cell lines inhibits tumorigenesis and metastasis in mice [54]. Importantly, elevated ATP7A expression was significantly associated with decreased survival in breast cancer patients, suggesting an important role for copper ion levels in human cancer metastasis. We have summarised the targets associated with copper ion transport and metabolism, along with their respective locations and functions within the cell. The results are presented in Table 1.

**TABLE 1:** Molecular targets related to copper metabolism and their primary functions and intracellular distribution.

Gene	Name	Function	Location
SLC31A1 (CTR1)	Solute Carrier Family 31 (Copper Transporter), Member 1	High‐affinity Cu importer	Plasma membrane
SLC31A2 (CTR2)	Solute Carrier Family 31 (Copper Transporter), Member 2	Adjust CTR1 function	Late endosome
ATP7A	ATPase Copper Transporting Alpha	Regulate intracellular copper homeostasis	Golgi apparatus; endosome; endoplasmic reticulum; plasma membrane
ATP7B	ATPase Copper Transporting Beta	Intracellular copper transport outward	Golgi apparatus; endosome; endoplasmic reticulum; mitochondrion
SCO1	Synthesis Of Cytochrome C Oxidase 1	Cytochrome c oxidase subunit II (MT‐CO2/COX2) is copper metallochaperone necessary for maturation.	Mitochondrion
SCO2	Synthesis Of Cytochrome C Oxidase 2	Regulate the redox state of cysteine in SCO1	Mitochondrion
COX11	Cytochrome C Oxidase Copper Chaperone	It may play its role in some terminal stages of cytochrome c oxidase synthesis by inserting copper B into subunit I.	Mitochondrion
SLC46A3	Solute Carrier Family 46 Member 3	Helps transport copper ions across the lysosome membrane	Lysosome; extracellular
ATOX1	Antioxidant 1 Copper Chaperone	Binding and delivery of cytosolic copper to copper ATPase proteins.	Cytosol; Golgi apparatus; nucleus; extracellular; plasma membrane
STEAP4	STEAP Family Member 1	Metalloreductase that has the ability to reduce both Fe(3+) to Fe(2+) and Cu(2+) to Cu(1+).	Endosome; plasma membrane
LOX	Lysyl Oxidase	Responsible for the post‐translational oxidative deamination of peptide lysine residues in collagen and elastin precursors	Extracellular; nucleus
FDX1	Ferredoxin 1	Key genes that regulate copper death	Mitochondrion
DLAT	Dihydrolipoamide S‐Acetyltransferase	Catalyses the full conversion of pyruvate to acetyl‐coa and carbon dioxide, linking glycolytic process to tricarboxylic acid cycle	Mitochondrion
LIAS	Lipoic Acid Synthetase	Binding with copper ions causes copper death	Mitochondrion

### 3.3 Disturbed Organelle Copper Homeostasis and Cancer Immunity

**FIGURE 3:**
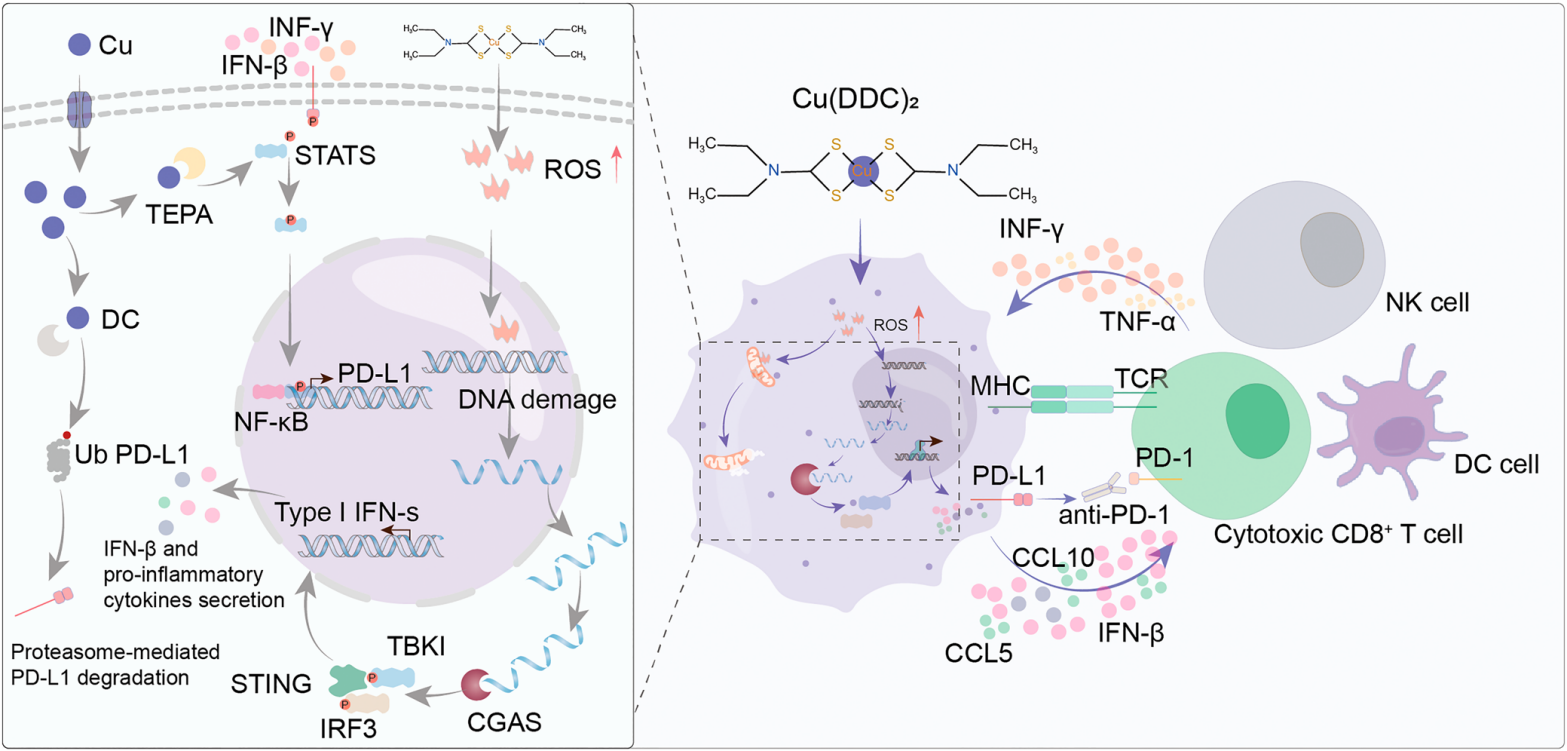
This figure illustrates the involvement of copper ions in the activation of the NF‐κB signalling pathway and the transcriptional process of PD‐L1 within cells. Copper ions enter the cells and promote cellular oxidative stress by increasing ROS production, leading to the release of inflammatory factors. Additionally, copper ions participate in inhibiting the ubiquitination process of PD‐L1 within the cells, thereby enhancing the stability of PD‐L1.

Tumour immune evasion is a significant factor affecting the efficacy of tumour immunotherapy. Therapeutic checkpoint antibodies that block programmed death receptor 1/programmed death ligand 1 (PD‐L1) signalling have greatly advanced cancer immunotherapy, achieving remarkable efficacy across various cancers. Recent studies indicate a significant correlation between copper ion content in tumour cells and PD‐L1 expression, highlighting its crucial role in mediating tumour immune escape [55]. Copper supplementation has been shown to enhance PD‐L1 expression at both mRNA and protein levels in cancer cells, with RNA sequencing revealing that copper regulates key signalling pathways driving PD‐L1‐mediated cancer immune escape. Conversely, copper chelating agents inhibit the phosphorylation of STAT3 and EGFR [31], promoting the ubiquitin‐mediated degradation of PD‐L1. Recent studies have demonstrated that a copper chelate formed from a terpyridine‐Cu complex can selectively target and bind to externalised phosphatidylserine on cancer cells. The copper chelators Dextran‐Catechin (DC) and TEPA inhibit the phosphorylation of STAT3 and EGFR, promote ubiquitin‐mediated degradation of PD‐L1, and significantly increase the number of tumour‐infiltrating CD8^+^ T cells and natural killer cells [31]. This interaction not only promotes the maturation of dendritic cells and the proliferation and infiltration of effector T cells into tumours, but it also significantly inhibits the expression of PD‐L1, thereby enhancing T cell‐mediated immune responses [56]. Regarding immune infiltration, copper chelating drugs significantly increase the number of tumour‐infiltrating CD8^+^ T cells and natural killer cells, slow tumour growth, and improve the survival rate of mice [31]. In contrast to copper chelation as a therapeutic strategy, some studies have utilised reactive oxygen species (ROS)‐sensitive nanoparticles loaded with copper chaperone inhibitors. By inhibiting the Atx1‐ATPase pathway, these nanoparticles promote significant accumulation of cisplatin and copper in cells, leading to considerable ROS production [57]. Excessive ROS induces intense endoplasmic reticulum (ER) stress, which promotes immunogenic cell death (ICD), thereby stimulating a sustained immune response. This approach also provides a novel concept and methodology for cancer immunotherapy. The detailed mechanism by which copper ions participate in anti‐tumour immunity is illustrated in Figure 3.

### 3.4 Disturbed Organelle Copper Homeostasis and Cancer Angiogenesis

In the process of tumour occurrence and development, numerous new blood vessels are formed. These new blood vessels supply the necessary nutrients and water for tumour growth [58]. Simultaneously, tumour cells can disseminate widely, leading to the formation of metastatic lesions in various parts of the body. As early as the late 20th century, the role of copper in angiogenesis was extensively elucidated [59]. However, the mechanisms by which copper participates in angiogenesis remained unclear at that time. At the beginning of the 21st century, it was demonstrated that copper deficiency could inhibit endothelial cell migration induced by exogenous FGF2 into the primitive vascular network [60, 61]. This study indicates that copper deficiency impedes tumour angiogenesis through two distinct mechanisms: first, by reducing the levels of extracellular angiogenesis‐promoting factors released by cancer cells, and second, by inhibiting endothelial cell differentiation. Furthermore, the study revealed that the copper ion chelator tetrathiomolybdate (TM) decreased the production of five angiogenic mediators in breast cancer, including vascular endothelial growth factor, fibroblast growth factor 2/basic fibroblast growth factor, interleukin‐1α, IL‐6 and IL‐8 [61]. It is also suggested that the primary mechanism underlying the anti‐angiogenic effect of TM‐induced copper deficiency is the inhibition of NF‐κB, which leads to a global suppression of the transcription of angiogenic factors mediated by NF‐κB. This view has been widely confirmed in subsequent studies. Although the mechanism between copper and angiogenesis is not very clear the anti‐angiogenesis strategy based on copper ion chelating agents has made a great breakthrough in guiding anti‐tumour drugs. In a recent study, it was observed that the copper chelating agent could reduce the activation of endothelial cells and normalise blood vessels in the treatment of mesothelioma tumours. The decreased copper content was also associated with CD4^+^ T cell infiltration [62]. Cu transporter antioxidant 1 (ATOX1) participates in Cu‐induced cell growth. It has been found that ATOX1 can participate in neointima formation after vascular injury by promoting VSMC migration and inflammatory cell recruitment in injured vessels [63]. This provides a new target and idea for anti‐tumour angiogenesis.

### 3.5 Disturbed Organelle Copper Homeostasis and Cancer Cell Death

The increase in copper ion concentration is closely associated with the proliferation of tumour cells. Recent research has unveiled a novel mode of cell death termed cuproptosis [5], which is intricately linked to copper ions within mitochondria. This study demonstrates that copper ions can induce the aggregation of lipoacylated proteins and oxidative stress, ultimately leading to cell death. There are two primary mechanisms underlying this mode of cell death [64]. Firstly, FDX1 facilitates the reduction of divalent copper to univalent copper, promoting the lipoacylation and aggregation of enzymes involved in the regulation of the mitochondrial TCA cycle, particularly DLAT. Secondly, FDX1 contributes to the instability of iron–sulfur (Fe‐S) cluster proteins. In addition to copper ion carriers, copper importers (such as SLC31A1) and exporters (such as ATP7B) modulate sensitivity to cuproptosis by influencing intracellular copper levels [24]. Cuproptosis is closely associated with alterations in mitochondrial enzyme function, as mitochondria serve as the primary site for copper‐induced cell death. Recent studies have corroborated this perspective by employing cells treated with copper ion carriers to monitor mitochondrial metabolites. The findings indicated that, with prolonged copper treatment, the disarray of numerous TCA cycle‐related metabolites intensified.

**FIGURE 4:**
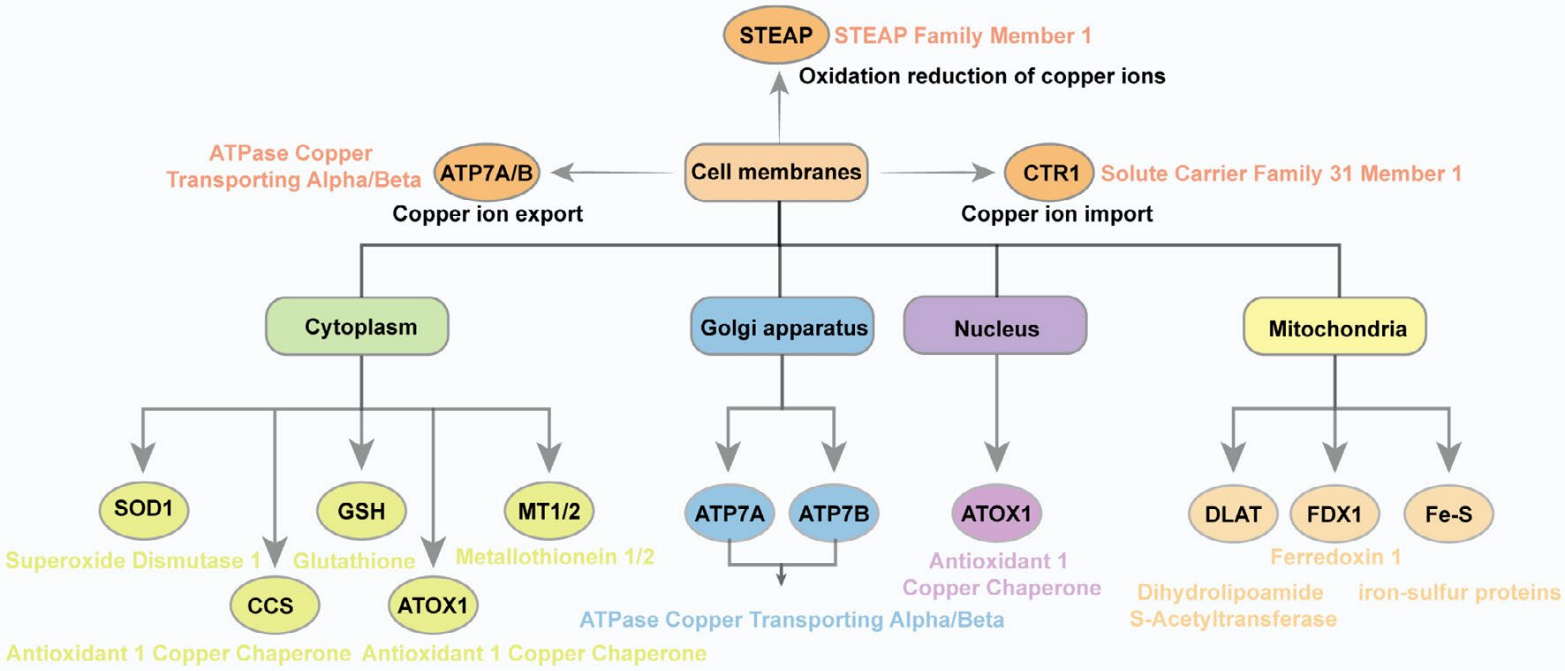
Copper ions transport and function in cells. Copper ions are transported into the cell via the membrane channel protein CTR1 after reduction by steap4 at the cell membrane. Within the cell, they are transported out of the membrane via vesicles or ATP7B. Some intracellular copper ions are transported toward the Golgi for storage via ATP7A/B. Most of the copper ions then function in the cytoplasm in combination with metabolic enzymes. Copper ions in mitochondria are involved in the synthesis of DLAT and the structural stability of iron–sulfur proteins, which in turn affects energy metabolism. ATOX1 carries copper ions to the nucleus and is involved in the activation of various signalling pathways.

Cuprotosis is not only associated with mitochondrial function but also plays a critical role in mitochondrial membrane dynamics. Inhibition of electron transport chain complexes I and II on the mitochondrial membrane significantly reduces copper‐induced cell death [65]. Furthermore, the mechanism underlying copper‐induced cell death involves the selective modification of various metabolic enzymes through lipoacylation. One notable acylated protein is dihydrothiamide S‐acetyltransferase (DLAT), a subunit of the pyruvate dehydrogenase complex. Copper directly interacts with DLAT, promoting disulfide bond‐dependent aggregation of fatty acylated DLAT, which leads to an inadequate supply of substrates for the tricarboxylic acid cycle. Additionally, mitochondrial ferredoxin (FDX1) and lipoyl synthase (LIAS) have been identified as crucial regulators in the process of copper‐induced cell death [20]. Gene knockout of either FDX1 or LIAS reduces protein lipoacylation and inhibits copper‐induced cell death [66]. The roles of these key genes in regulating copper‐induced cell death in cancer have been extensively documented, particularly the close relationship between FDX1 and the prognosis of various cancers. The detailed distribution and functional annotation of these molecules are illustrated in Figure 4.

Altered copper homeostasis is a significant factor in the progression of various diseases, particularly neurodegenerative disorders. Numerous studies have demonstrated a direct correlation between disrupted copper homeostasis and the advancement of several neurodegenerative diseases, including Alzheimer's disease (AD), Huntington's disease (HD) and amyotrophic lateral sclerosis (ALS) [67]. The most comprehensive research has focused on AD, where elevated levels of copper have been identified in senile plaques and serum of patients. Furthermore, in mouse models of AD, high concentrations of copper were observed in Aβ plaques within the dentate gyrus subregion of the hippocampus [68]. Notably, copper chelation therapy and the knockdown of the copper transporter protein CTR1 have been shown to mitigate neurotoxicity in a Drosophila model of AD [31]. These findings underscore the critical role of copper in the progression of neurodegenerative diseases, highlighting its significance in the onset and development of these conditions. The potential mechanisms underlying neurodegenerative diseases share similarities with copper‐induced cell death. For instance, the exposure of neuroblastoma cells to copper markedly increased mitochondrial reactive oxygen species (ROS) production, subsequently leading to a reduction in pyruvate dehydrogenase levels within the tricarboxylic acid (TCA) cycle [49]. This phenomenon is a crucial aspect of cuproptosis. Nevertheless, to ascertain whether copper toxicity contributes to the pathogenesis of neurodegenerative diseases and whether the inhibition of copper toxicity could serve as a novel therapeutic strategy for patients suffering from these debilitating and progressive disorders, further research is clearly warranted.

The dual role of copper in tumour development is noteworthy. On one hand, elevated copper levels can promote tumour growth by inducing the production of reactive oxygen species (ROS), exacerbating genomic instability and influencing various tumour‐associated signalling pathways [69]. Conversely, excessive copper can lead to tumour cell death. When copper levels are overloaded, the initial consequence is the production of substantial amounts of reactive oxygen species, which play a crucial role in inducing pyroptosis [70]. Overloaded copper preferentially activates CASP1, which cleaves gasdermin D (GSDMD) into its N‐ and C‐terminal components, facilitating the maturation and release of inflammatory cytokines such as interleukin‐1β (IL‐1β). The N‐terminal domain of GSDMD subsequently binds to the cell membrane, forming oligopores that result in water influx and cell death [71]. In the non‐classical pyroptosis pathway, CASP4/5/11 can be activated by intracellular lipopolysaccharides (LPS), leading to the cleavage of GSDMD into its N‐terminal domain and promoting pyroptosis. Copper‐ion overload influences various components of the phosphatidylinositol‐3‐kinase (PI3K)‐AKT signalling pathway, leading to downstream activation [72]. Specifically, copper can directly activate PI3K, which in turn activates AKT. Additionally, copper binds to histidine residues 117 and 203 of pyruvate dehydrogenase kinase 1 (PDK1) [31], resulting in AKT activation. This copper‐induced activation of AKT further catalyses the phosphorylation and subcellular redistribution of FoxO1a and FoxO4, thereby promoting cancer cell proliferation and tumour growth. Furthermore, the activation of the mitogen‐activated protein kinase (MAPK) signalling pathway is also contingent upon the presence of copper ions; copper directly binds to mitogen‐activated protein kinase 1 (MEK1) [31, 73], facilitating the phosphorylation of ERK1/2 and activating the downstream c‐Jun N‐terminal kinase (JNK), which regulates tumour growth. Additionally, AKT activation has been shown to further activate the downstream mTOR pathway, accelerating the formation of the BECN1‐PIK3C3 complex, which is critical for the initiation of cellular autophagy [20].

**FIGURE 5:**
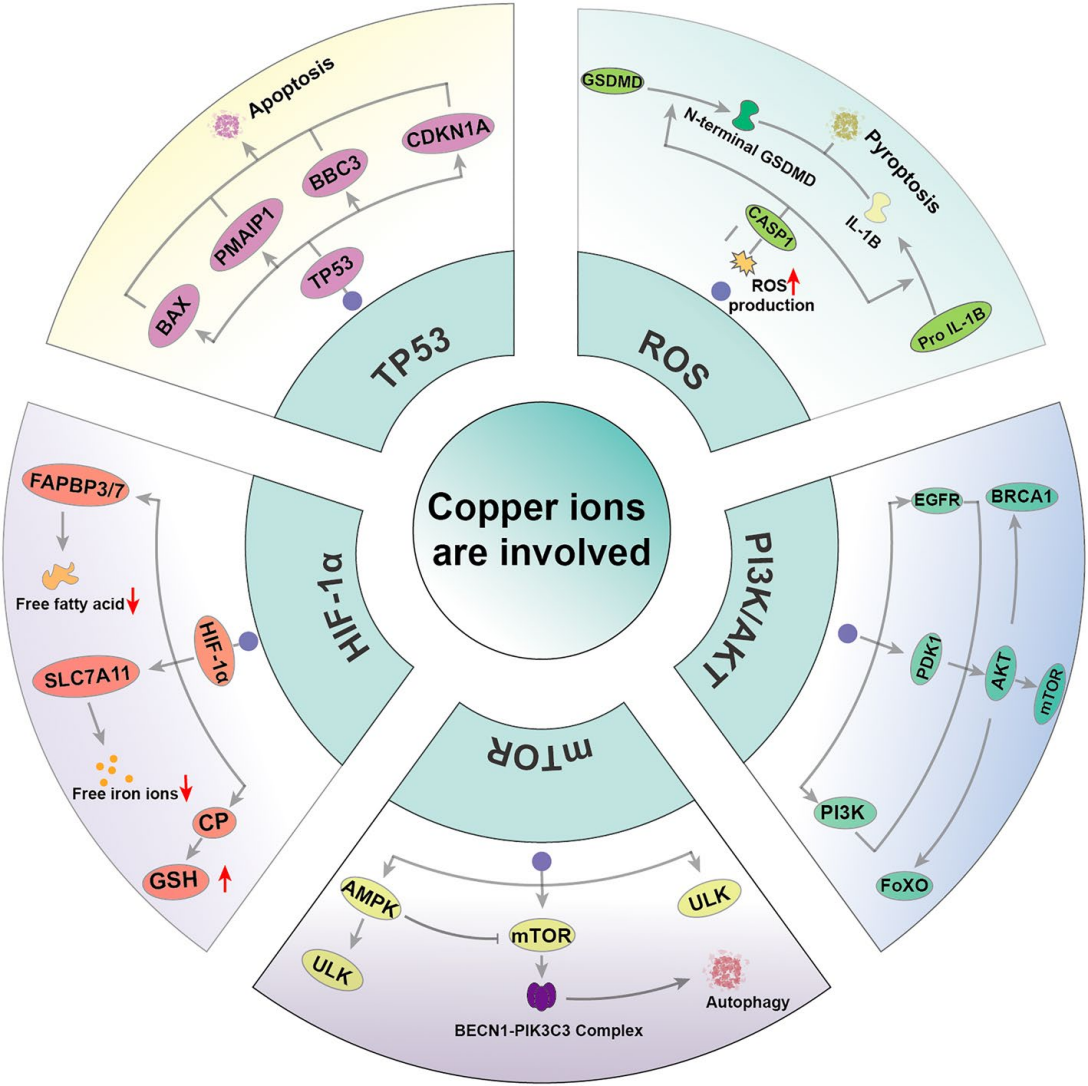
Copper is closely associated with the activation of cancer signalling pathways. Copper overload induces cellular pyroptosis by increasing the production of reactive oxygen species, which in turn induces cellular pyroptosis. In addition, it can also induce the process of apoptosis by activating TP53. Activation of mTOR through PI3K/AKT causes the onset of the cellular autophagy process. The occurrence of the cellular ferroptosis process can likewise be induced by affecting HIF‐1α.

Numerous reports have indicated that copper ions play a critical role in tumour angiogenesis, which is contingent upon the interaction between copper and the hypoxia‐inducible factor 1 subunit α (HIF‐1α)‐related signalling pathways [20]. Copper facilitates the binding of HIF‐1α to essential motifs in the promoters of target genes in a CCS‐dependent manner, thereby upregulating the expression of affected genes, including hypoxia‐inducible factor 1 (HIF1) [74]. Even in normoxic conditions, copper can directly enhance the stability of HIF‐1α, promoting the expression of target genes such as vascular endothelial growth factor (VEGF), which contributes to tumour angiogenesis. Furthermore, copper promotes HIF1A stabilisation, leading to increased transcription of HIF1A target genes, including FABP3, FABP7, CP and SLC7A11 [75]. This copper‐mediated upregulation of HIF1A target genes inhibits lipid peroxidation and ferroptosis. Similarly, TP53 serves as a crucial regulator of apoptosis through the transcription of various apoptotic target genes. The activation of the TP53 pathway is implicated in copper‐induced apoptosis. Reports indicate that in human breast cancer MCF7 cells [76], excess copper ions elevate the expression of TP53, which subsequently enhances the expression of BAX. This cascade results in the opening of the mitochondrial permeability transition pore and the generation of reactive oxygen species (ROS). Other TP53 target genes, including p21 (cyclin‐dependent kinase inhibitor 1A) [77], PMAIP1/NOXA (phorbol‐12‐myristate‐13‐acetic acid‐induced protein 1), and BBC3/PUMA (BCL2‐binding component 3), collectively regulate copper‐induced apoptosis. The evidence regarding the aforementioned copper ion‐activated pathways promoting tumour progression has been compiled and summarised in Figure 5.

## 4 Therapeutic Value of Manipulating Cu^2+^ Homeostasis in Tumour Cells

### 4.1 The Relationship Between Copper Transporter and Cellular Drug Resistance

Increasing our understanding of intracellular copper signalling networks has significantly advanced drug design and clinical applications. Despite rapid advancements in tumour immunotherapy, chemotherapy remains the most commonly employed treatment for cancer [78]. However, drug resistance among patients is a significant factor that impedes the therapeutic efficacy of chemotherapy agents. Therefore, elucidating the comprehensive mechanisms of drug action may be a crucial strategy to enhance chemotherapy effectiveness and improve patient prognosis [79]. The association between copper and resistance to cancer chemotherapy has been recognised for an extended period. Previous studies have primarily concentrated on the critical role of copper ion‐dependent proteins in mediating anti‐tumour drug resistance [80, 81]. Recent investigations have revealed that lysosomes play a pivotal role in mediating drug resistance in tumour cells [82]. Several copper transporters are instrumental in lysosome‐mediated cisplatin resistance [83, 84]. Notably, human copper transporter 1 (HCTR1) has been demonstrated to be associated with the uptake of platinum‐based antineoplastic agents, with HCTR1 overexpression leading to increased cisplatin accumulation in various tumour cell line models [85]. Consequently, patients exhibiting undetectable levels of CTR1 showed diminished platinum accumulation and reduced tumour response [86]. Thus, HCTR1 is crucial in platinum resistance and is regarded as a predictive marker for pre‐treatment evaluation in patients with non‐small‐cell lung carcinoma.

The copper export transporter ATP7B plays a crucial role in lysosomal‐mediated resistance to cisplatin [87]. As a copper export transporter, the loss of ATP7B function leads to excessive copper accumulation in cells, resulting in copper poisoning [88]. Recent studies indicate that ATP7B translocates from the Golgi apparatus to the lysosome, promoting lysosomal copper accumulation in the presence of excess copper within the cellular microenvironment [89]. Furthermore, ATP7B has been shown to facilitate lysosomal exocytosis, which aids in the removal of copper from cells, thereby mitigating copper poisoning. The transport of copper within cells appears to be interconnected with the transport of cisplatin [90]. Specifically, the overexpression of ATP7B in prostate carcinoma cells correlates with decreased cisplatin accumulation, contributing to cisplatin resistance [91]. Most studies highlight the significance of ATP7B translocation in the development of metal resistance to chemotherapeutic agents. When chemotherapy drugs, such as cisplatin, enter the cell, ATP7B is relocated from the Golgi apparatus to the lysosome and vesicles, effectively excluding metal ions, including cisplatin, from the cell via exocytosis. This mechanism renders cancers less sensitive to chemotherapy during treatment.

In addition to the two channel proteins responsible for copper transport, Copper Transporter 2 (CTR2) has also shown a significant association with antitumor drug resistance [31]. Unfortunately, few studies have focused on the critical role of this copper transporter in cells, highlighting a promising research direction. It has been found that CTR2 is structurally similar to CTR1 and is predominantly located on the lysosomal and plasma membranes of tumour cells [92]. However, despite their structural similarities, CTR2 and CTR1 exhibit opposing effects on cisplatin resistance in cancer cells. Specifically, the knockdown of CTR2 increases the influx of cisplatin into cells, resulting in enhanced cisplatin cytotoxicity, whereas overexpression of CTR2 has been shown to confer increased resistance to cisplatin [92, 93]. There are two prevailing theories regarding the role of CTR2 in cisplatin resistance. The first posits that CTR2 expression is crucial for maintaining the stability of CTR1. In this context, it has been demonstrated that CTR2 induces the production of a truncated form of CTR1 that lacks its extracellular domain, which is less efficient at transporting cisplatin within tumour cells. The second theory suggests that CTR2 regulates the rate of macropinocytosis by activating Rac1 and Cdc42, thereby influencing the transport of cisplatin [94]. These two mechanisms may collectively impact the accumulation of cisplatin and copper within cells. However, despite the significant role of this copper transporter in tumour angiogenesis and drug resistance, studies elucidating the mechanisms of CTR2 remain relatively sparse.

### 4.2 Anti‐Tumour Strategy by Improving Copper Homeostasis

An important aspect of the application of copper homeostasis in cancer treatment is the development and use of copper ion chelators. Given the elevated copper levels observed in various cancers, copper ion chelating agents, which aim to reduce these levels, have increasingly become a significant component of cancer chemotherapy [94]. The anti‐tumour activity of copper ion chelators has been recognised since the mid‐20th century. The most widely utilised copper ion chelator is tetrathiomolybdate (TM), which has demonstrated considerable efficacy in the treatment of breast cancer and head and neck cancer [71, 95]. The primary mechanism of TM involves the reduction of copper levels and the inhibition of the nuclear transcription factor (NF‐κB). NF‐κB is closely associated with tumour growth, metastasis and angiogenesis, thereby enabling TM to exert a beneficial anti‐tumour effect [20]. TM is generally well tolerated, exhibiting minimal side effects at conventional doses. However, it is important to note that copper plays a vital role in normal haematopoiesis, and its reduction may impact this process. Fortunately, this effect is readily reversible with a decrease in TM dosage. Overall, TM offers a novel perspective for the development of anti‐tumour drugs by enhancing copper homeostasis in tumour cells.

Disulfiram (DSF) is an FDA‐approved drug for the treatment of alcoholism. It acts as an inhibitor of acetaldehyde dehydrogenase, which oxidises ingested alcohol into acetaldehyde, leading to the accumulation of acetaldehyde in the body [20, 96]. The resulting acetaldehyde can further combine with proteins and nucleic acids, establishing an alcohol aversion reflex. Recently, researchers have discovered that DSF also demonstrates promising results in anti‐tumour therapy. Specifically, DSF can serve as a modulator to reverse tumour multidrug resistance. Studies indicate that DSF influences the cellular localisation of efflux transporter ATPase, such as ATP7A [97]. When administered in conjunction with certain chemotherapy drugs, DSF enhances the sensitivity of tumour cells to these agents. Notably, when DSF is combined with cisplatin, it inhibits NF‐κB activation by reducing intracellular levels of glutathione (GSH) [97, 98], which in turn regulates apoptosis‐related proteins, thereby improving the efficacy of cisplatin against drug‐resistant cancer cells. Furthermore, GSH levels are negatively correlated with the cuproptosis process in cells, as GSH binds to copper in mitochondria, alleviating the cuproptosis process [99]. However, the relationship between DSF and copper‐induced cell death appears to have been overlooked in the existing literature.

**FIGURE 6:**
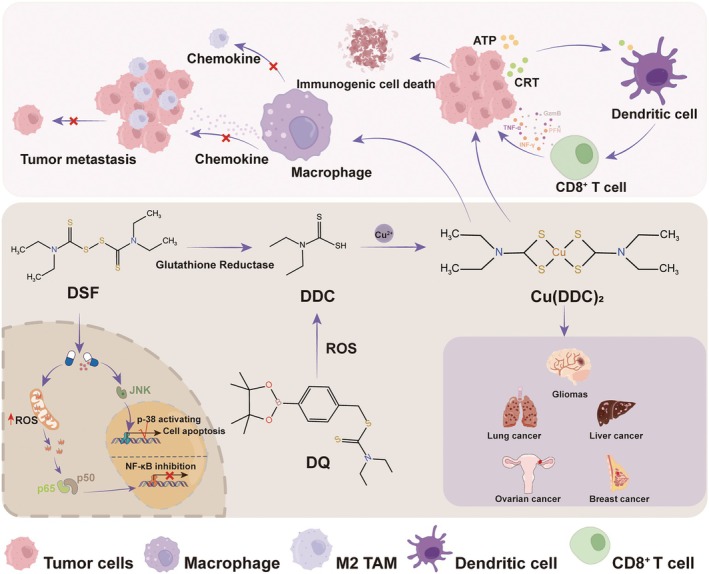
Drugs designed based on copper ions affect immune function. DSF is Glutathione converted into DDC in cells, where two DDC molecules bind to one copper ion to form Cu (DDC)_2_. Cu (DDC)_2_ can not only influence macrophage differentiation, thereby preventing metastasis of tumour cells, but also enable CD8 + T cells to secrete cytokines, leading to tumour cell immunogenic cell death.

Through the use of patient‐derived xenotransplantation models, it has been demonstrated that the combination of disulfiram (DSF) and cisplatin exhibits a more significant therapeutic effect than the application of either drug alone. Furthermore, DSF itself serves as an effective anti‐tumour agent. Diethyl dithiocarbamate (DDC), a metabolite of DSF in vivo, can form a complex with copper ions (Cu^2+^) that possesses anti‐tumour activity [100]. The accumulation of this complex can lead to protein degradation, polyubiquitination and the immobilisation of p97/NPL4, ultimately resulting in apoptosis [101]. The anti‐tumour effect of DSF is dependent on Cu^2+^; however, the concentration of Cu^2+^ in the human body is exceedingly low, which significantly diminishes the anti‐tumour efficacy of DSF. Consequently, recent studies have explored the combination of DSF with copper nanoparticles for cancer treatment. Notably, copper sulfide nanoparticles (CuS NPs) have been widely utilised as photothermal therapeutic agents in conjunction with chemotherapy and photothermal therapy based on DSF [102]. Copper sulfide nanoparticles (CuS NPs) not only interact with disulfiram (DSF) metabolites in vivo to exert anti‐tumour effects, but they also demonstrate exceptional photothermal conversion capabilities. These nanoparticles can inhibit tumour progression through a combination of chemotherapy and photothermal therapy, thereby achieving a synergistic therapeutic effect [103]. This method increases intracellular copper levels to induce copper toxicity, leading to cell death and the destruction of tumour cells. With the discovery of cuproptosis, the strategy of inducing tumour cell death by elevating copper levels within cells presents promising prospects. Dithiocarbamate (DQ) is an aminomethyl dithiocarbamate derivative that generates dithiocarbamate (DDC) in response to reactive oxygen species (ROS) [104]. The chelation of copper and DDC further generates ROS, initiating a cascade of oxidative stress. Additionally, DQ‐derived methoquinone (QM) targets glutathione (GSH) vulnerability; this effect, combined with increased ROS levels, disrupts redox homeostasis and leads to cancer cell death. Importantly, the formation of Cu(DDC)_2_ serves as a potent cytotoxic anticancer agent that effectively induces immunogenic cell death (ICD) [104]. The synergistic effects of epithelial‐mesenchymal transition (EMT) modulation and ICD are crucial for managing cancer metastasis and potential drug resistance. The mechanism of action of DSF is illustrated in Figure 6.

### 4.3 The Impact of Iumor Heterogeneity on Targeted Copper Ion Therapy Strategies

The hypoxia‐inducible factor HIF‐1α upregulates the expression of the copper transporter CTR1, promoting the uptake of copper by cancer cells [57]. Cancer cells in hypoxic regions may be more sensitive to cuproptosis, but it should be noted that HIF‐1α also promotes the expression of the copper efflux protein ATP7A, which can lead to the development of drug resistance [103]. The acidic tumour microenvironment (pH 6.5–6.8) can activate pH‐sensitive nanocarriers (such as Cu‐ZIF‐8) to target the release of copper ions, increasing local concentration and reducing systemic toxicity [105]. However, the acidic environment may enhance the transmembrane transport capacity of copper ionophores (such as Elesclomol), but excessive acidification can compromise the integrity of the cell membrane, leading to non‐specific copper leakage [48]. Cancer cells with active glycolysis (Warburg effect) resist cuproptosis by upregulating ATP7B to efflux copper, whereas mitochondria‐dependent cells are more susceptible to cuproptosis due to copper accumulation. This may suggest that combining glycolysis inhibitors (such as 2‐DG) could promote cuproptosis, thereby enhancing therapeutic efficacy. Cancer‐associated fibroblasts (CAFs) secrete TGF‐β, inducing CTR1 expression in cancer cells to promote copper uptake; meanwhile, CAFs themselves protect neighbouring cancer cells by delivering copper chelators (such as metallothioneins) via exosomes [51]. This may indicate that targeting the CAFs‐TGF‐β signalling pathway could enhance the targeting of cuproptosis‐related therapeutic strategies. In summary, the heterogeneity of the tumour microenvironment profoundly influences the efficacy of copper ion‐targeted therapeutic strategies by regulating copper uptake, efflux and metabolic pathways. In the future, it will be necessary to integrate single‐cell sequencing, spatial transcriptomics and live imaging technologies to decipher the spatiotemporal dynamics of copper metabolism within the TME, and to develop intelligent delivery systems for precise intervention.

## 5 Conclusions and Perspectives

Numerous studies have established a connection between copper homeostasis and cell proliferation, tumour growth and metastasis [2, 69]. However, the limited research on the underlying mechanisms hinders our ability to elucidate the causal relationships among these processes. There is a notable deficiency in studies linking copper‐dependent targets and pathways to tumour vulnerability. Furthermore, the distribution and storage mechanisms of copper within cells lack sufficient evidence, which impedes our understanding of the dynamic transport processes of copper [21]. The valence transformation and localization of copper in cells are critical for its biological function. Therefore, it is essential to continue developing novel chemical probes to monitor unstable copper, particularly with respect to subcellular resolution and oxidation state specificity, as well as the capability to conduct comparative analyses of total copper and unstable copper states across different cell populations. Recent research on copper‐based nanomaterials (Cu‐based NMs) has demonstrated significant potential in tumour therapy, attributed to their strong near‐infrared absorption, high photothermal conversion efficiency and good biocompatibility, which render them suitable for photothermal therapy (PTT) and photodynamic therapy (PDT) [106, 107]. Moreover, their targeting capability can be enhanced through surface modification. However, the preparation and application of Cu‐based NMs may generate wastewater and exhaust gases, potentially posing threats to environmental safety. Furthermore, Cu‐based NMs have not yet been utilised in actual clinical practice due to insufficient exploration of their long‐term biosafety, pharmacokinetics and immunoreactivity [31]. Therefore, it is imperative to thoroughly assess their safety and toxicity through extensive and rigorous mammalian testing. Nevertheless, with ongoing breakthroughs and innovations in drug synthesis, it is anticipated that these limitations will be effectively addressed in the near future.

With the concept of cuproptosis proposed, the application of targeted copper homeostasis in cancer treatment emerges as a promising field. However, due to the low content of copper in the human body, its involvement in various life activities poses challenges [20]. While targeting copper homeostasis therapy for cancer, it often inadvertently disrupts other biological processes. Therefore, enhancing the specificity of cuproptosis represents a critical challenge that must be addressed. Numerous studies have demonstrated the advantages of targeted copper delivery in cancer treatment. For instance, pH‐dependent copper release is achieved by encapsulating copper complexes within pH‐sensitive long‐circulating liposomes, providing a novel approach to developing pH‐sensitive nanotherapy strategies for metal‐based drugs in colon cancer treatment [103, 106]. Nanoliposomes containing copper complexes exhibit significant anti‐cancer effects and safety, not only in rectal cancer but also in melanoma. The focus extends beyond merely altering copper homeostasis in tumour cells; it also encompasses strategies targeting organelle‐specific copper homeostasis for cancer treatment [106]. For example, employing organelle‐specific copper depletion, particularly in nanoparticles that modify mitochondrial copper homeostasis, has garnered considerable attention for its potential in cancer therapy. This strategy enhances specificity and mitigates off‐target effects. Studies indicate that both copper depletion and supplementation to toxic levels are viable strategies for manipulating copper poisoning pathways.

The cellular distribution of copper ions is complex and primarily functions within mitochondria. Therefore, understanding the relationship between mitochondrial copper transport and other organelles holds significant importance for cancer treatment. It is now widely accepted that the imbalance of copper homeostasis is not merely a by‐product of cancer progression, but rather a driving factor in various contexts. This situation arises due to dysregulated copper regulators and persistent tumour‐driving events. The discovery of copper‐induced cell death further underscores the necessity of expanding our understanding of the mechanisms regulating copper homeostasis, which can lead to this reverse cellular outcome. Although numerous unresolved issues persist in this field, a comprehensive understanding of the regulatory mechanisms of copper homeostasis in cancer represents a frontier in the development of innovative approaches to cancer treatment.

## Author Contributions

**Chengxin Chen:** conceptualization (equal), writing – original draft (equal), writing – review and editing (equal). **Mengle Peng:** conceptualization (equal), visualization (equal), writing – original draft (equal), writing – review and editing (equal). **Shuhong Liang:** conceptualization (equal), writing – original draft (equal), writing – review and editing (equal). **Chunwei Li:** writing – original draft (equal), writing – review and editing (equal). **Lili Zhu:** writing – review and editing (equal). **Yaqi Yang:** conceptualization (equal), writing – original draft (equal). **Lifeng Li:** writing – review and editing (equal). **Wenhua Xue:** conceptualization (equal), funding acquisition (equal), writing – review and editing (equal).

## Conflicts of Interest

The authors declare no conflicts of interest.

## Data Availability

All data is publicly available.
